# 583. Real-world Utilization of Serological tests in Patients with Suspected Scrub typhus in South Korea: A Single-center, Observational study

**DOI:** 10.1093/ofid/ofad500.652

**Published:** 2023-11-27

**Authors:** SeulKi Kim, A Reum Kim, Seungjin Lim, Su Jin Lee, Moonsuk Bae

**Affiliations:** Pusan National University Yangsan Hospital, Yangsan-si, Kyongsang-namdo, Republic of Korea; Pusan National University Yangsan Hospital, Yangsan-si, Kyongsang-namdo, Republic of Korea; Pusan National University Yangsan Hospital, Yangsan-si, Kyongsang-namdo, Republic of Korea; Pusan National University Yangsan Hospital, Yangsan-si, Kyongsang-namdo, Republic of Korea; Pusan National University Yangsan Hospital, Yangsan-si, Kyongsang-namdo, Republic of Korea

## Abstract

**Background:**

The most widely used diagnostic methods for scrub typhus are serological tests. We investigated the utilization of serological tests for scrub typhus in clinical practice and evaluated their clinical usefulness.

**Methods:**

The data of all adult patients with suspected scrub typhus were collected who visited a tertiary-care hospital during the autumn season (between September and December) of the years 2019, 2020, and 2021. All included patients had an acute fever (axillary temperature 37.5) and had at least one of the following ten secondary findings: myalgia, nonspecific skin rash, eschar, headache, thrombocytopenia, increased liver enzymes, lymphadenopathy, hepatomegaly, splenomegaly, pleural effusion. Clinical diagnosis was independently adjudicated by two infectious diseases physicians and finally classified into two groups (1) scrub typhus and (2) other diseases. Two commercially available serological testing for scrub typhus were performed.

**Results:**

We evaluated 172 patients with suspected scrub typhus; of them, 136 met the eligibility criteria, including 109 (33%) who were diagnosed with scrub typhus and 27 (67%) who were diagnosed with other diseases. The clinical characteristics between the groups are shown in Table 1. Single, paired total antibodies using immunofluorescence assay (IFA) testing, and total antibodies using immunochromatography (ICA)-based rapid diagnostic testing (RDT) were measured in 98%, 22%, and 75% of all patients, respectively (Table 2). The final laboratory confirmation using paired samples for scrub typhus was established at a median of 10 (IQR 9-15) days following the first visit day. The median follow-up time of all patients was 13 (IQR 8-19) days. The discrepancy between ICA results and clinical diagnosis was 26% for the scrub typhus group and 33% for the other disease group.

**Figure:**
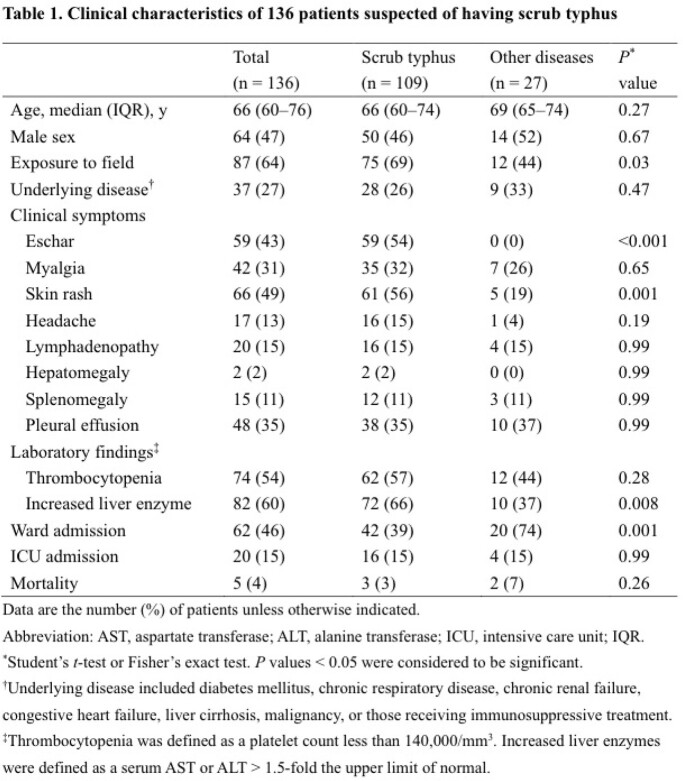

**Figure:**
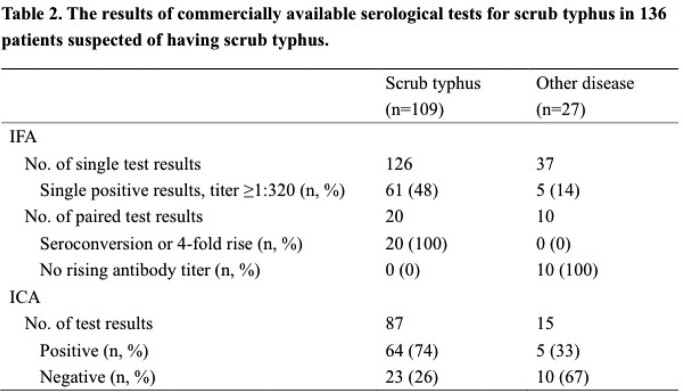

**Conclusion:**

Serological testing currently used for scrub typhus in clinical practice in South Korea had limitations in clinical decision-making for scrub typhus.

**Disclosures:**

**All Authors**: No reported disclosures

